# The Effect of Hydroxy Silicone Oil Emulsion on the Waterproof Performance of Cement

**DOI:** 10.3390/ma17122797

**Published:** 2024-06-07

**Authors:** Xuewen Quan, Fen Zhou, Chaocan Zhang, Shuangping Ma

**Affiliations:** 1 School of Materials Science and Engineering, Wuhan University of Technology, Wuhan 430070, China; 317254@whut.edu.cn (X.Q.); 245184@whut.edu.cn (F.Z.);; 2 Wuhan Subo New Building Materials Co., Ltd., Wuhan 430082, China

**Keywords:** hydroxy silicone oil emulsion, polyether-modified silicone oil, integral hydrophobic modification

## Abstract

The hydrophilic and porous structure of cement-based concrete materials makes it vulnerable to various harmful ions dissolved in water in the environment or during the freeze–thaw cycle, resulting in a significant decline in durability. Therefore, the introduction of hydrophobic hydroxyl silicone oil with good chemical stability and excellent hydrophobic properties during the process of concrete preparation to achieve the hydrophobic modification of its internal holes has very positive significance in terms of improving its durability. In order to disperse the hydrophobic hydroxyl silicone oil evenly in the internal pores of the concrete, synthetic non-ionic polyether-modified silicone oil was used as an emulsifier to make it a water-soluble emulsion. The influences of the composition of the emulsifier on the dispersion, water contact angle, water absorption, porosity, and compressive strength of cement mortar were investigated. The results show that when the emulsion content is 0.5%, the pore volume of the cement mortar decreases by 15%, and the maximum contact angle reaches 128°, which is conducive to improving the anti-erosion and anti-freezing properties of concrete and provides a new solution for the preparation of high-durability concrete. However, the introduction of polyether-modified silicone oil increases the number of large holes in the cement mortar, and leads to an increase in water absorption and a decrease in compressive strength. It is necessary to further optimize the composition of emulsifier in future work.

## 1. Introduction

Cement-based materials, known for their high mechanical strength and durability, serve as the cornerstone of modern construction and infrastructure development. They are extensively utilized in various transportation engineering projects such as roads, bridges, railways, tunnels, dams, and civil engineering structures [1]. However, the presence of numerous gel pores, capillary pores, and macropores within the structure of cement-based materials renders them highly hydrophilic [2], leading to issues such as frost damage [3], shrinkage cracking, alkali–aggregate reactions, and reinforcement corrosion [4].Aggressive ions such as sulfate ions [5], chloride ions [6], magnesium ions, and other corrosive ions in the environment infiltrate the internal pores of cement-based materials, facilitated by water as a carrier and medium. This ion infiltration causes the aging and cracking of cement-based materials, resulting in a decrease in their service life. According to statistics, the annual infrastructure maintenance cost due to concrete corrosion exceeds USD 100 billion [7], causing significant economic losses to the national economy and severe damage to the natural environment.

In recent years, researchers have turned their attention to the hydrophobic or superhydrophobic modification of cement-based materials, inspired by microstructures found in nature such as lotus leaves and cicada wings, which possess low surface free energy and high surface roughness [7,8]. Through hydrophobic modification, the surfaces of cement-based materials transition from hydrophilic to hydrophobic, resulting in reduced water permeability and decreased vulnerability to the damaging effects of erosive ions, thus enhancing durability. Hydrophobic modification can be categorized into surface modification and bulk modification [9], with the former involving the creation of a hydrophobic protective layer through methods like coating [10,11,12,13] or impregnation [14,15], and the latter achieved by adding hydrophobic additives to impart overall hydrophobic properties.

Ray [11] developed a superhydrophobic coating using a hexadecyltrimethoxysilane (HDTMS)-solution-treated silica sol. Jiang [16] devised two negative carbon superhydrophobic self-cleaning concrete coatings by incorporating a combination of silane- and silicon-carbide-modified recycled concrete powder (RP). Szymańska [15] utilized a hydrophobic impregnation technique to convert concrete specimens from hydrophilic to hydrophobic. While surface hydrophobic modification can enhance the impermeability of cement-based materials, it is unable to prevent the infiltration of gases or vapors. Moreover, surface hydrophobic coatings tend to lose adhesion and easily detach upon contact with moisture during use, resulting in a significant reduction in their waterproofing effectiveness, mechanical properties, and service life.

In contrast, the incorporation of hydrophobic admixtures during the mixing process for integral hydrophobic modification proves to be more effective in extending the service life of cement-based materials. The most common hydrophobic admixtures include liquid silanes and siloxanes [17], carbon powders [18], stearic acid emulsions [19], and silane emulsions. These hydrophobic admixtures are uniformly dispersed on the surface and within the internal pores of cement-based materials, resulting in an increased contact angle and improved impermeability. Falchi [20] introduced hydrophobic admixtures containing calcium stearate, zinc stearate, and silane/siloxane into concrete, demonstrating excellent hydrophobic performance. Zhu [21] employed silane for the integral hydrophobic modification of recycled concrete, resulting in enhanced durability but a 41% reduction in compressive strength, possibly due to the dispersion of hydrophobic admixtures within the pores affecting the nucleation and growth of hydration products. Mora [22] incorporated hydrophobic silica particles into mortar, achieving a contact angle of 122° and a 45% reduction in water absorption, with a 6% decrease in compressive strength. Wang [23] reported a decrease in water absorption from 11% to 1% in modified mortar samples using polydimethylsiloxane as a hydrophobic admixture, with reductions of 31% in compressive strength and 18% in flexural strength. Wu [24] found that incorporating 1% calcium stearate emulsion into cement mortar resulted in a 51% reduction in water absorption after 48 h, with a contact angle of 130°. After 28 days, the compressive and flexural strength of the mortar decreased by 15% and 14%, respectively.

Organosilicon compounds have high chemical stability and good hydrophobic, acid, alkali, salt, and weather resistance. Its chemical inertness prevents it from participating in the hydration reaction of cement and destroying the composition of the hydration products. Therefore, to improve the overall waterproof performance and durability of cement mortar, this study adopts hydroxy-terminated silicone oil as the hydrophobic polymer of concrete, and synthesizes non-ionic silicone surfactants using the silohydroaddition method. The commonly used OP-7 emulsifier is used as the reference sample. The influences of emulsion system and formulation on the water contact angle, setting time, dispersion, porosity, water absorption, and mechanical strength of cement mortar were investigated, which provided research ideas for improving the durability of concrete.

## 2. Materials and Methods

### 2.1. Materials

The polyoxyethylene alkyl ether was supplied by Hangzhou Danwei Co., Ltd. (Hangzhou, China). The hydrosiloxane oil was provided by Zhejiang Runhe Organic Silicon New Materials Co., Ltd. (Ningbo, China). Chloroplatinic acid and isopropanol were supplied by Guoyao Chemical Reagent Co., Ltd. (Shanghai, China). The hydroxyl-terminated silicone oil was provided by Wuhan Huaxiang Kechie Biological Technology Co., Ltd. (Wuhan, China). OP-7 was supplied by Shanghai Lianji Chemical Co., Ltd. (Shanghai, China). Deionized water was used, and the cement used was in accordance with the ISO 679-2009 standard [25] cement provided by Fushun Cement Co., Ltd. (Fushun, China). The sand used was in accordance with the ASTM C33 standard [26] sand provided by Xiamen ESUN standard sand Co., Ltd. (Xiamen, China).

### 2.2. Synthesis of Hydroxyl-Terminated Silicone Oil Emulsifier

Under a nitrogen atmosphere, a certain amount of polyoxyethylene alkyl ether and a trace amount of chloroplatinic acid catalyst dissolved in isopropanol solution were added to a 250 mL four-necked flask equipped with an electric stirrer and a thermometer. The stirrer was started, and the reaction temperature was strictly controlled at 110 °C. After a period of time, a certain amount of hydrosiloxane oil was added dropwise into the four-necked flask using a burette, maintaining the set reaction temperature. The reaction proceeded for 8 h to obtain polyether-modified silicone oil (PMSO) [27,28].

### 2.3. Preparation of Hydroxyl-Terminated Silicone Oil Emulsion

Under ice-water bath conditions, hydroxyl-terminated silicone oil, OP-7, PMSO, and a mixture of OP-7 and PMSO emulsifiers were separately weighed according to the prescribed ratios and placed into three-necked flasks. The mechanical stirrer speed was adjusted to 300 rpm and stirred for 30 min. After the allotted time, a certain amount of deionized water was added. The mechanical stirrer speed was then increased to 3000 rpm and continued stirring for 6 h. Upon completion of stirring, the mixture was poured into a beaker and further homogenized at high speed for 0.5 h using a homogenizer. Finally, seven different ratios of hydroxyl-terminated silicone oil emulsions were obtained.

### 2.4. Preparation of Cement Slurry

Deionized water and cement were quickly measured and poured into a mixing pot using a cement slurry mixer. The mixer was started, and low-speed stirring was carried out for 120 s, followed by a 15 s pause. During this pause, the cement slurry on the blades and walls of the pot was scraped into the center of the pot. Subsequently, high-speed stirring was conducted for 120 s before stopping the machine.

### 2.5. Preparation of Cement Mortar

Seven types of cement mortar were prepared with a 0.5% dosage of the respective hydroxyl-terminated silicone oil emulsions. The mixing ratio of cement to sand was 1:3 by mass, with a water/cement ratio of 0.4, and the quantities used were 450 g each. The mechanical and impermeability properties of the mortar were tested after adding the homemade emulsions using the following method: The hydroxyl-terminated silicone oil emulsion, water, and cement were sequentially added to a JW900 mixer from Shandong Hengtuo Machinery Technology Co., Ltd. (Jining, China). Slow stirring was conducted for 30 s, followed by a pause for 30 s, during which standard sand was automatically added. Rapid stirring was then performed for 60 s to obtain the cement mortar. For the blank control group, the amount of water was adjusted to 190 and the resulting cement mortar was poured into different molds. After curing in an environment at a temperature of 20 ± 3 °C and a relative humidity (RH) of 90%, the specimens were demolded and tested at different ages. Specimens with dimensions of 70.7 mm × 70.7 mm × 70.7 mm were used for water absorption rate testing, while specimens measuring 40 mm × 40 mm × 160 mm were used for compressive strength and other parameter testing.

### 2.6. Measurement and Characterization

#### 2.6.1. Measurement of Hydrophobic Modifier’s Physicochemical Properties 

##### Cloud Point, HLB Value, Self-Emulsification Capability, and PH Measurement

The cloud point was determined according to the method specified in ISO 1065:1991 [29] for the determination of the cloud point index (in water) of nonionic surfactants. The HLB value was determined using the modified titration method outlined by H.L. Greenward. The self-emulsification capability was tested according to the standard with a mass fraction of 40%. The pH value was measured following the method outlined in ISO 23496:2019 [30] for the determination of pH values of chemical reagents.

##### Viscosity and Particle Size Measurement

The viscosity of the hydrophobic modifier was measured using an NDJ-5S rotary viscometer (Drawell, Chongqing, China). The particle size of the hydrophobic modifier was analyzed using a Bettersize 3000 laser diffraction particle size analyzer (Bettersize Instruments, Chongqing, China).

#### 2.6.2. Characterization of Hydrophobic Modifier Structure

A small amount of emulsion sample was dissolved in CDCl_3_ and placed in an NMR tube for ^1^H NMR measurement. An Agilent 400 M nuclear magnetic resonance spectrometer (Agilent, Santa Clara, CA, USA) was used to test the ^1^H NMR resonance spectra of the samples. The solvent used was deuterated chloroform without the addition of TMS.

#### 2.6.3. Characterization of Cement Mortar Structure

Standard-cured cement mortar samples were crushed into small pieces after being cured for 28 days, and the central portion was ground into powder. Fourier-transform infrared spectroscopy (FTIR) and X-ray photoelectron spectroscopy (XPS) were used to characterize the structure of the cement mortar.

#### 2.6.4. Testing of Cement Mortar Workability and Mechanical Properties 

##### Standard Consistency and Setting Time Testing of Cement Mortar

The standard consistency and setting time of the cement mortar were measured according to ISO 9597:2008 [31] using a DL-AWK Vicat apparatus.

##### Flowability Testing of Cement Mortar

Cement mortar was prepared according to GB/T 8077-2023 [32], and then the hydrophobic silicone oil emulsion, water, cement, and sand were added to the mixing bucket in the proportions described above. After mixing for 2~3 min, the flowability of the cement mortar was measured using an NLD-3 flowability tester according to GB/T 2419-2005 [33].

##### Compressive Strength Testing of Cement Mortar

The compressive and flexural strengths of the cured cement mortar samples were tested using a WTS-type mortar compressive and flexural testing machine following ISO 679:2009.

#### 2.6.5. Evaluation of Hydrophobic Properties of Cement Mortar

##### Water Contact Angle Measurement

The water contact angle of the cut surface of the cured cement mortar samples (size: 40 mm × 40 mm × 160 mm) was measured using a JY-PHb contact angle meter after cleaning with deionized water and drying.

##### Water Absorption Testing

The water absorption of the cured cement mortar samples (size: 70.7 mm × 70.7 mm × 70.7 mm) was tested according to the standard method outlined in JC474-2008 [34].

#### 2.6.6. Pore Structure Analysis of Cement Mortar

The pore structure of the cured cement mortar was analyzed using a BSD-660M pore size analyzer (Beishide Instrument Technology (Beijing) Co., Ltd., Beijing, China).

## 3. Results and Discussion

### 3.1. Analysis of Synthesis of Hydroxyl-Terminated Silicone Oil Emulsifiers

**Figure 1:**
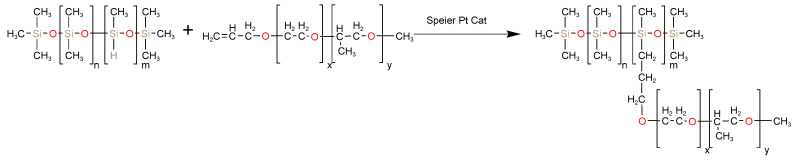
Hydrosilane addition reaction equation.

Due to the difficulty of emulsifying silicone oil and the significant impact of ionic emulsifiers on cement hydration [35,36,37], we synthesized a series of nonionic surfactants containing siloxane to enhance the stability of the silicone oil emulsions using the principle of similar solubility. Five different ratios of polyether-modified silicone oil were synthesized according to the chemical reaction equations shown in Figure 1. The raw material ratios and product properties are shown in Table 1.

According to the data in the table, the molar ratio of ethylene to silane in the synthesis of polyether-modified silicone oil is 1.0:1.2. The resulting HLB value is 8.2, with a cloud point of 45 °C. It easily self-emulsifies, forming stable light blue micelles with a particle size of 63 nm. The research suggests that the excessive hydrophilicity of the emulsifier may have a negative impact on the hydration of the cement. However, when the HLB value is at the critical state between hydrophilic and hydrophobic, its influence on cement hydration may be minor. Therefore, we selected the polyether-modified silicone oil synthesized at a ratio of 1.0:1.2 as the organic silicon emulsifier.

### 3.2. Structure Analysis of Hydroxy-Terminated Silicone Oil Emulsifier

**Figure 2:**
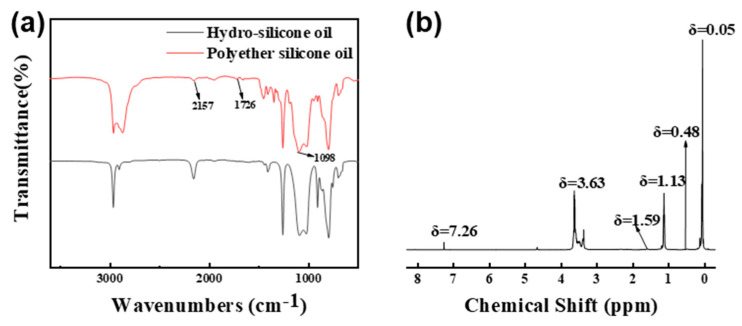
(**a**) Infrared characteristics of hydrogen-containing silicone oil and polyether-modified silicone oil; (**b**) ^1^H NMR images of polyether-modified silicone oil.

Figure 2a shows the infrared spectrum of the silicone oil containing hydrosilane and the synthesized polyether-modified silicone oil. In the spectrum of the silicone oil containing hydrosilane, a strong absorption peak of Si-H appears at 2157 cm^−1^, while the characteristic absorption peak at the same position in the synthesized product is significantly weaker, indicating the involvement of Si-H in the reaction. The appearance of the C=C stretching vibration absorption peak at 1726 cm^−1^ confirms the participation of vinyl-terminated polyoxyethylene ether in the reaction. The infrared characteristic peak at 1098 cm^−1^ corresponds to the stretching vibration of Si-O-Si, further confirming the hydrosilylation reaction. Based on the above analysis, it is evident that the Si-H in the hydrosilane reacts with the C=C of the vinyl-terminated polyoxyethylene ether, and the Si-H is replaced by Si-C. Figure 2b shows the proton nuclear magnetic resonance spectrum of the polyether-modified silicone oil, with δ = 7.29 corresponding to the solvent peak of CDCl_3_. In the spectrum of the product, no characteristic absorption peak corresponding to the silicon–hydrogen bond at δ = 4.89 is observed, while characteristic absorption peaks appear at δ = 3.30~3.72, indicating that the hydrosilylation reaction has occurred. Proton absorption peaks near δ = 0.48 correspond to protons in the silicon methyl group, those near δ = 1.07~1.24 correspond to protons in the submethyl group of the polyether-modified silicone oil, and δ = 3.63 ppm corresponds to the proton absorption peak of -CH_2_-O- in the polyether, indicating that the -CH_2_-O- bond acts as a side-linking branch to the silicone oil containing hydrosilane. The nuclear magnetic resonance spectrum further confirms that the system has undergone the hydrosilylation reaction, yielding the target product of polyether-modified silicone oil.

### 3.3. Preparation and Performance Analysis of Hydroxy-Terminated Silicone Oil Emulsion

**Figure 3:**
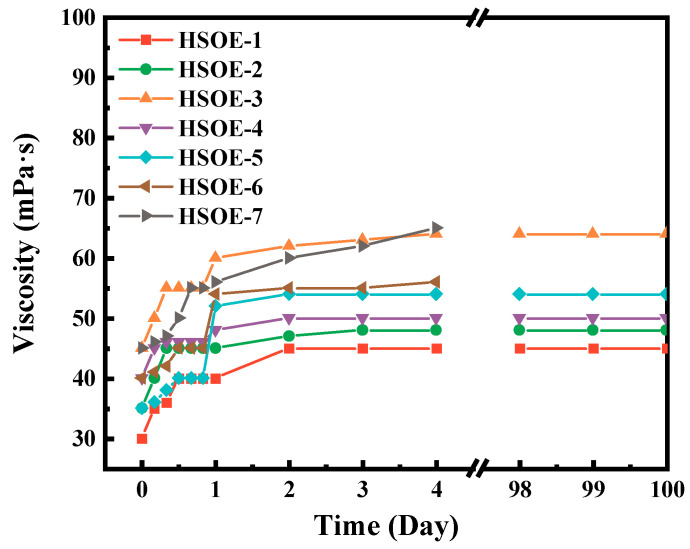
Emulsion viscosity diagram of different hydroxyl silicone oils.

Based on the experiments described above, Table 2 shows seven different hydroxyl-terminated silicone oil emulsions (referred to as HSOEs hereafter) were prepared using various emulsifiers and proportions. The sample with PMSO accounting for 30% of the hydroxyl-terminated silicone oil exhibited the smallest particle size and the highest viscosity. The viscosity of all samples changed to varying degrees within 96 h and stabilized after 48 h. The test results for samples 1–3 from Figure 3 showed that with increasing emulsifier content, the viscosity gradually increased, and the particle size decreased accordingly. This is because a higher concentration of emulsifier leads to stronger dispersion, resulting in a reduction in emulsion particle size. Samples 1, 4, 5, 6, and 7 revealed that the introduction of OP-7 into the system caused it to disperse into the continuous phase under mechanical action, interacting with the polyether silicone oil, thereby reducing the stability of the emulsion and increasing the particle size. OP-7 exhibits lower hydrophobicity compared to PMSO. Therefore, with constant usage, an increase in OP-7 content led to a decrease in the proportion of PMSO, resulting in decreased emulsion stability. Consequently, the particle size of samples 3–7 increased sequentially.

### 3.4. Influence of Hydroxy-Terminated Silicone Oil Emulsion on the Structure of Cement Mortar

**Figure 4:**
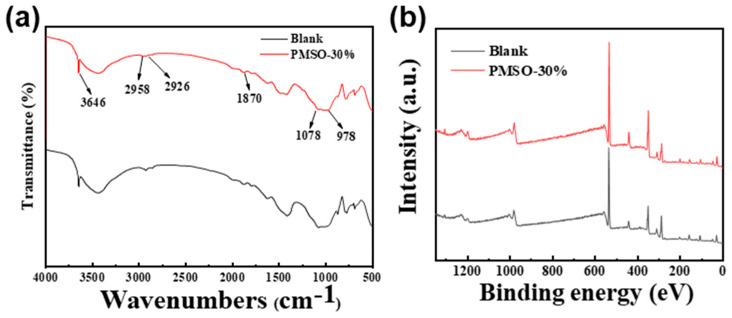
(**a**) Infrared spectra of unmodified and HSOE-3-modified cement mortar. (**b**) X-ray photoelectron spectroscopy.

Figure 4a shows the infrared characteristic spectra of unmodified and HSOE-3-modified cement mortar. In the figure, distinct differences can be observed in the absorption peaks of the unmodified and modified cement mortar in certain bands. The absorption peak at 3646 cm^−1^ corresponds to the asymmetric stretching vibration of hydroxyl groups [38]. The peaks at 2958 cm^−1^ and 2926 cm^−1^ correspond to the asymmetric and symmetric stretching vibrations of -CH_2_, respectively. The stretching vibration peak of Si-O appears at 1078 cm^−1^, while there is no vibration peak at this wavelength for the blank sample 1 [39]. The infrared spectroscopic analysis results indicate that the hydroxyl-terminated silicone oil is distributed in the modified cement mortar.

Figure 4b and Table 3 present the X-ray photoelectron spectroscopy (XPS) spectra of unmodified and HSOE-3-modified cement mortar, showing that both the unmodified and modified cement mortar surfaces contain the elements C, O, Ca, and Si [40]. The analysis reveals that the carbon element originates from emulsion additives, the calcium element from cement hydration products and mortar samples, the silicon element from emulsion additives and cement hydration products, and the oxygen element from emulsion additives and cement hydration products. This indicates the presence of hydroxyl-terminated silicone oil emulsion on the surface of the modified cement mortar, further confirming the successful addition of hydroxyl-terminated silicone oil emulsion to the cement mortar. Combined with the infrared spectra, it can also be inferred that the hydroxyl-terminated silicone oil emulsion can disperse on the internal and external surfaces of the cement mortar.

### 3.5. Hydroxy Silicone Oil Emulsion Dispersion Properties of Cement Mortar and Performance Analysis of Net Cement Slurry Setting Time

#### 3.5.1. Dispersion

**Figure 5:**
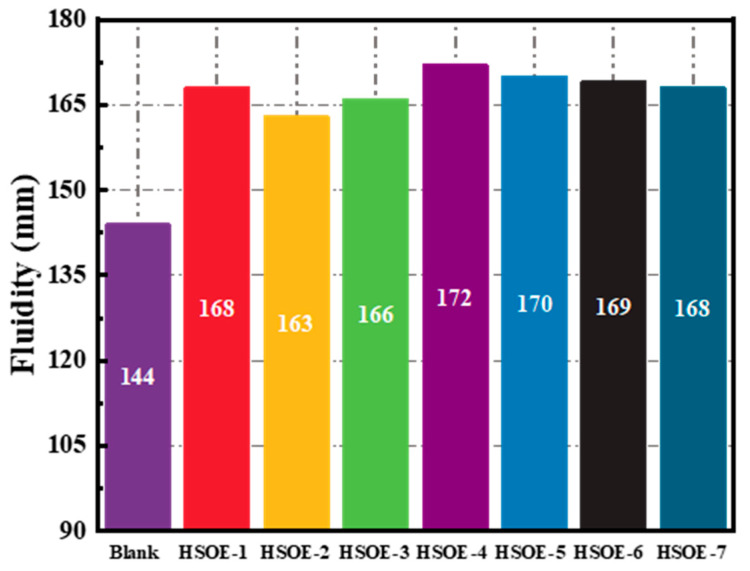
The influence of different emulsion-modified cement slurry on the fluidity of mortar.

Figure 5 illustrates the influence of different HSOEs on the fluidity of cement mortar modified with HSOEs. The fluidity of all cement pastes with added hydroxyl-terminated silicone oil emulsion is significantly higher than that of the blank reference sample, indicating a noticeable dispersion effect of all emulsions on cement particles. Hydroxyl-terminated silicone oil possesses both hydrophilic hydroxyl groups (-OH) and hydrophobic silicon–oxygen bonds (Si-O). The -OH groups can adsorb onto the surface of cement particles, while the hydrophobic nature of the Si-O bonds reduces the surface tension of cement particles, thereby increasing the fluidity of the cement paste. The differences in the fluidity of cement paste with added hydroxyl-terminated silicone oil emulsion are small, indicating that the type and amount of emulsifier have little effect on the fluidity of the cement paste, i.e., the emulsifiers used do not have a significant dispersion effect on cement particles.

#### 3.5.2. Influence on Setting Time of Clean Pulp

**Figure 6:**
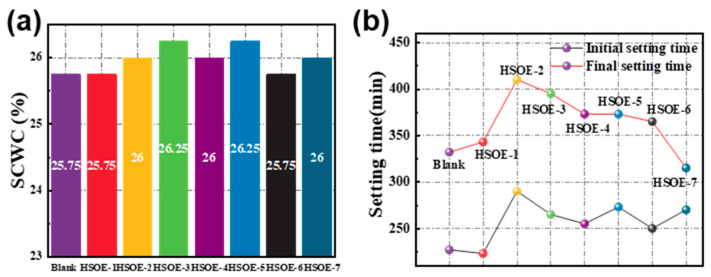
Influence of different emulsions on (**a**) water consumption of standard consistency (SCWC) and (**b**) setting time of clean slurry.

Figure 6a,b demonstrate the influence of different HSOE-modified cement pastes on the water requirement for the standard consistency and setting time of the paste. From Figure 6a, it can be observed that the water requirement for the standard consistency of all cement pastes containing HSOEs is similar to that of the blank reference group, indicating that the addition of HSOEs has almost no effect on the water requirement for standard consistency of cement paste. In Figure 6b, the initial and final setting times of the blank reference sample are 227 min and 332 min, respectively. For the HSOE-1 emulsion prepared with 10% PMSO, the initial and final setting times are almost unchanged at 223 min and 343 min, respectively, compared to the blank reference sample. As the PMSO content increases from 10% to 20%, the initial and final setting times increase to 290 min and 410 min, respectively, indicating a significant delay in cement hydration with increasing PMSO content, which is related to the ability of silane to inhibit cement hydration [41]. PMSO is a silane polymer containing a large number of ether bonds, and when it is enveloped on the surface of cement particles, it can hinder further contact between cement particles and water, leading to a significant inhibition of hydration, manifested by a significant extension of the setting time [42]. When the dosage further increases from 20% to 30%, the initial and final setting times no longer increase, but slightly decrease. This may be because the PMSO has saturated the surface of the cement particles, and the excess PMSO can only enter the cement pore solution, and its inhibitory effect on hydration no longer continues to increase. Unlike PMSO, OP-7 is a nonionic surfactant with a limited inhibitory effect on cement hydration.

**Figure 7:**
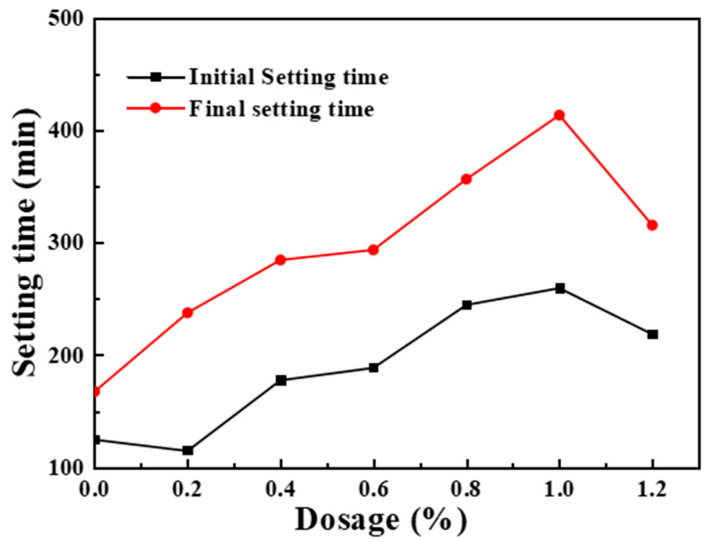
Effect of PMSO content on setting time of cement slurry.

The above analysis shows that the synthesized PMSO has a delaying effect on cement hydration. Then, we separately analyzed the rule of influence of PMSO content on setting time, and the results are shown in Figure 7. In the range of 1.0%, with the increase in the PMSO content, the initial and final setting time of the cement slurry was gradually extended. When the dosage increased from 1.0% to 1.2%, the initial and final setting time of cement slurry decreased significantly. The results further confirmed that the inhibition effect of HSOE on cement hydration came from PMSO.

### 3.6. Analysis of Hydroxy Silicone Oil Emulsion on the Properties of Cement Mortar

#### 3.6.1. Effects on Cement Mortar Water Contact Angle

**Figure 8:**
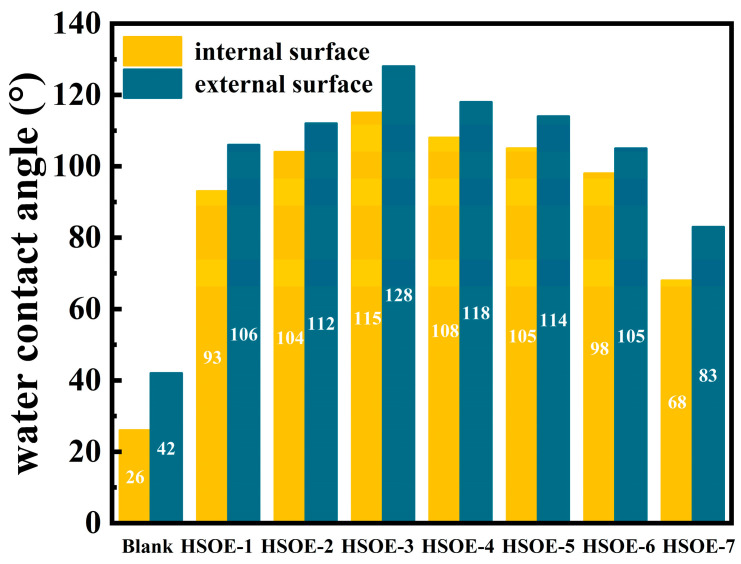
Influence of different emulsion-modified cement mortar on water contact angle.

Figure 8 illustrates the influence of seven different formulations of HSOE on the water contact angle of cement mortar on both internal and external surfaces. The internal and external surface contact angles of the blank reference sample are only 26° and 42°, respectively, while the water contact angles on both internal and external surfaces of all HSOE-containing cement mortars are significantly higher than those of the blank reference sample. With the increase in the PMSO content, the water contact angles of the cement mortar on both internal and external surfaces rapidly increase, reaching 115° and 128°, respectively, when 30% PMSO is added. As the amount of OP-7 replacing PMSO increases, the contact angles of the mortar on both internal and external surfaces decrease successively, and the emulsion prepared with pure OP-7 has a smaller effect on the water contact angle of the mortar compared to the emulsion prepared with pure PMSO. These results indicate that hydroxy-terminated silicone oil exhibits excellent hydrophobicity, and the hydrophobic effect of emulsions prepared with PMSO is significantly better than for those prepared with OP-7. The smaller the particle size of the silicone emulsion, the larger the surface area of the emulsion particles, the more uniform the dispersion of the hydrophobic material, the greater the number of silicone particles filling the pores of the cement mortar, and the better the hydrophobic effect.

#### 3.6.2. Influence on Water Absorption of Cement Mortar

**Figure 9:**
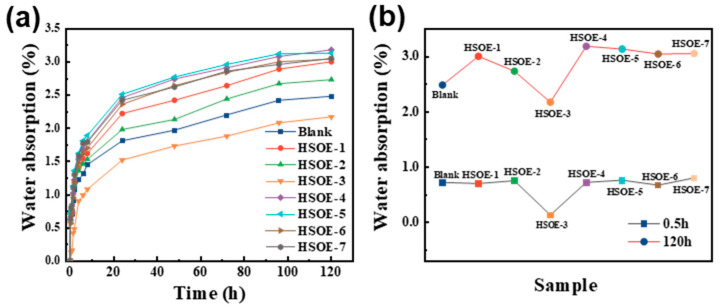
(**a**) Curve of water absorption over time; (**b**) curve of water absorption over time for 0.5 h and 120 h modified cement mortar.

Figure 9 depicts the variation curve of the water absorption rate over time for cement mortar modified with different HSOE formulations. The water absorption rate of the blank reference sample cement mortar at 120 h is 2.5%, while the water absorption rate of cement mortar containing 30% PMSO reaches 2.2% at 120 h, representing a decrease of 12.5%. This reduction could be attributed to the presence of 30% PMSO in HSOE-3, which causes significant changes in pore size distribution when the emulsion is added to the cement mortar, resulting in a decrease in pore volume and size, thus affecting the mortar’s water absorption rate. However, as the amount of co-added OP-7 increases, the water absorption rate of the mortar gradually increases at 120 h.

#### 3.6.3. Influence on Folding Strength and Compressive Strength of Cement Mortar

**Figure 10:**
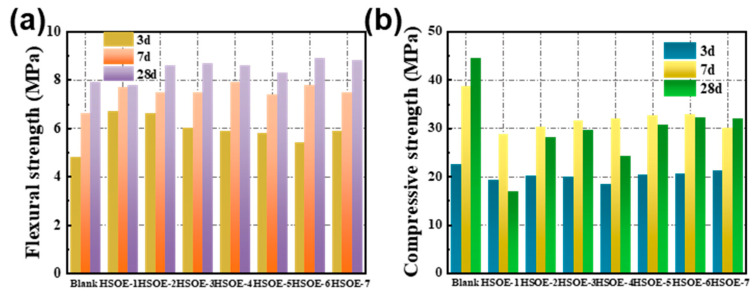
The mechanical strength of cement mortars with different hydroxy-silicone oil emulsions at different ages: (**a**) flexural strength; (**b**) compressive strength.

Figure 10a,b illustrate the compressive strength and flexural strength of cement mortar modified with different HSOE formulations at different ages. From the figures, it can be observed that, at the same age, the compressive strength of all cement mortars containing HSOE is lower than that of the blank reference group, while the flexural strength is the opposite, being higher than the blank reference group. The compressive strength and flexural strength of the blank reference group at 28 days are 44.6 MPa and 7.9 MPa, respectively. For the cement mortar with 30% PMSO added, the compressive strength and flexural strength are 29.7 MPa and 8.7 MPa, respectively. The compressive strength decreases by 34%, while the flexural strength increases by 10%. As can be seen from Table 4, with the extension of time, the compressive strength of cement mortar at 140 d is significantly higher than that at 28 d, which is closer to the blank group, and the strength loss at 140 d becomes smaller and smaller, which may be related to the slight influence of the emulsifier and emulsion prepared by us on cement hydration in the early stage, but it will not prevent the hydration of cement in the later stage.

Compressive strength and flexural strength refer to the maximum pressure and stress that cement mortar can withstand under compression and bending, respectively. The former depends on the compression strength between particles and molecules, while the latter is determined by the bonding force between different particles and molecules. The hydration of cement mortar is hindered after the addition of HSOE, inhibiting the nucleation and growth of hydration products such as calcium silicate hydrate (CSH gel), ettringite (AFt), and calcium hydroxide (CH), which inevitably affects the compressive strength of cement mortar at different ages. In addition, the pore structure of cement mortar changes correspondingly after the addition of HSOE, which may also be the main reason for the change in compressive strength.

Hydroxy-terminated silicone oil can form silane polymers through intermolecular dehydration. Under the encapsulation effect of silane polymers, the bonding force in the interface transition zone between cement paste and sand is correspondingly enhanced, leading to an increase in flexural strength of all cement mortars containing HSOE. OP-7 has a smaller inhibitory effect on cement hydration than PMSO, and PMSO has an encapsulation effect. Therefore, as the proportion of OP-7 replacing PMSO increases, the impact on compressive strength and flexural strength becomes more significant. Moreover, it is worth noting that, except for the blank reference group, almost all cement mortars containing HSOE have lower compressive strength at 28 days than that at 7 days, which needs to be taken into consideration.

#### 3.6.4. Influence on the Pore Structure of Cement Mortar

**Figure 11:**
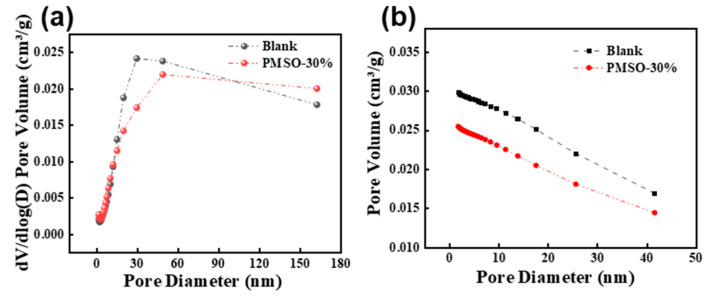

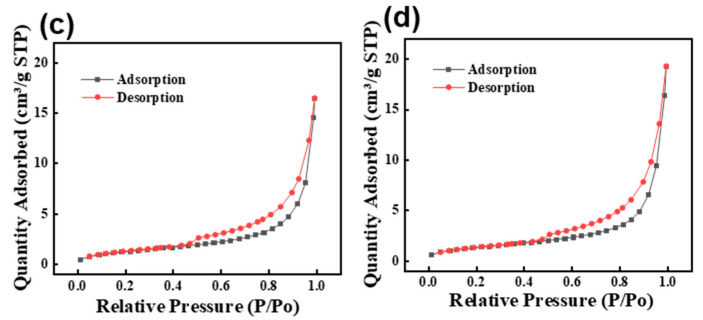
(**a**) Differential curve of cement mortar pore size distribution; (**b**) integral curve of cement mortar pore size; (**c**) adsorption isotherm of cement mortar blank reference sample; (**d**) adsorption isotherm of cement mortar mixed with 30% PMSO.

Figure 11a illustrates the differential curve of pore size distribution of cement mortar. The maximum pore volume of the unmodified mortar is 0.029804 cm^3^/g, while that of the cement mortar modified with 30% PMSO is 0.025478 cm^3^/g. This indicates that the silane polymers generated by the reaction of hydroxy-terminated silicone oil with water fill the pores of the cement mortar, resulting in a corresponding decrease in pore volume, which is consistent with the significant decrease in cumulative pore volume shown in Figure 11b. Figure 11c represents the pore size distribution of unmodified and HSOE-3-modified cement mortar samples, showing that the pore size thresholds of the blank reference and cement mortar modified with 30% PMSO are 30 nm and 49 nm, respectively. After adding HSOE, the porosity of the cement mortar is lower than that of the blank reference group, while the pore size threshold increases, indicating that the volume of relatively small pores decreases while the volume of larger pores increases. This suggests that HSOE introduces a certain number of large bubbles. HSOE contains PMSO with an HLB value of only 8.2, which can reduce the surface tension of the pore solution when added to cement mortar. This may be the reason why adding HSOE can reduce porosity but increase the pore size threshold. This may also be one of the main reasons for the increase in the mortar water absorption rate and the decrease in compressive strength.

## 4. Conclusions

The present study successfully synthesized non-ionic organosilicone surfactants as emulsifiers using the hydrosilylation method and prepared emulsions containing hydroxyl-terminated silicone oil (HSOE) as hydrophobic additives for cement mortar, utilizing them along with OP-7 and other emulsifiers. The effects on cement hydration, contact angle, mechanical properties, etc., were investigated, leading to the following conclusions:

1. Polyether-modified silicone oil with an HLB value of 8.2 and turbidity point of 45 °C was prepared by using a vinyl polyoxyethylene ether and silicon hydrogen bond molar ratio of 1.0:1.2, which has excellent self-emulsifying properties and is a stable light blue micelle with a particle size of 63 nm. Then, five kinds of hydroxy-terminated silicone oil emulsions were prepared by using it with OP-7, but with the increase in OP-7, the stability of the hydroxy-terminated silicone oil emulsions gradually deteriorated.
2. HSOE has a good hydrophobic effect, which can significantly improve the contact angle of cement mortar, in line with expectations, and the smaller the particle size of emulsion and the more uniform the dispersion of hydrophobic substances, the better the hydrophobic effect.
3. PMSO has a significant delay effect on the early stage of cement hydration, but it does not prevent the later stage of cement hydration, and with the extension of time, the loss of compressive strength gradually decreases compared with the blank group.
4. In the future, an emulsifier that can emulsify hydroxyl silicone oil and has no effect on cement hydration can be selected. The influence of the emulsifier on the stability of water-based emulsion can be further discussed, and a long-term durability experiment could be carried out to study the influence of emulsion on shrinkage and long-term stability.

## Figures and Tables

**Table 1:** Properties of five different polyether-modified silicone oils.

Samples	Raw Material Ratio	Product Property					
	n(C=C): n(Si-H)	HLB	Cloud Point (°C)	pH	Viscosity (mpa·s)	Micellar Morphology	Micelle Size (nm)
PMSO-1	1.5:1.0	11.2	51	6.8	2100	Reflex Blue	53
PMSO-2	1.0:1.0	9.4	48.5	7.2	2300	Reflex Blue	61
PMSO-3	1.0:1.2	8.2	45	7.3	2600	Reflex Blue	63
PMSO-4	1.0:1.4	7.5	41.5	7.3	2800	Reflex Blue	65
PMSO-5	1.0:2.0	6.3	36	7.5	3000	Light white	93

**Table 2:** Properties of different hydroxy-silicone oil emulsions.

Sample	Emulsifier Species	Emulsifier Content	Hydroxy-Terminated Silicone Oil Concentration (%)	Emulsion Viscosity (mPa·s)	Emulsion Particle Size (nm)	Emulsion Stability
HSOE-1	PMSO-3	10%	40	45	1064	Stabilization
HSOE-2	PMSO-3	20%	40	48	965	Stabilization
HSOE-3	PMSO-3	30%	40	64	872	Stabilization
HSOE-4	PMSO-3:OP-7 = 3.5:0.5	10%	40	50	1130	Stabilization
HSOE-5	PMSO-3:OP-7 = 3.0:1.0	10%	40	54	1651	Stabilization
HSOE-6	PMSO-3:OP-7 = 2.0:2.0	10%	40	56	2601	Precipitation after 85 days
HSOE-7	OP-7	10%	40	65	3800	Precipitation after 75 days

**Table 3:** Distribution of different element contents (%) on the surface of unmodified and HSOE-3-modified cement mortars.

Element	UCM	MCM
C	35.98	27.88
O	42.8	45.86
Ca	14.69	21.48
Si	6.53	4.78

**Table 4:** Comparison table of compressive strength of 28 d and 140 d cement mortar.

Sample	28 Days of Compressive Strength (MPa)	140 Days of Compressive Strength (MPa)
Blank	44.6	47.8
HSOE-1	16.9	20.7
HSOE-2	28.2	30.6
HSOE-3	29.7	32
HSOE-4	24.2	27.3
HSOE-5	30.7	32.4
HSOE-6	32.3	34.9
HSOE-7	32	38.6

## Data Availability

Data are contained within the article.
